# Pulsatile intraspinal pressure drives post-traumatic syringomyelia: evidence supporting the CSF pulsatile pressure dynamic equilibrium hypothesis

**DOI:** 10.1186/s12987-026-00846-x

**Published:** 2026-08-13

**Authors:** Chenghua Yuan, Fan Yuan, Chao Chang, Jiachen Wang, Zuowei Wang, Xingwen Wang, Hao Wu, Zan Chen, Fengzeng Jian, Jian Guan

**Affiliations:** 1 Department of Neurosurgery, Xuanwu Hospital, Capital Medical University, Beijing, China; 2 Spine Center, China International Neuroscience Institute (CHINA-INI), Beijing, China; 3 Research Center of Spine and Spinal Cord, Beijing Institute of Brain Disorders, Capital Medical University, Beijing, China; 4 Lab of Spinal Cord Injury and Functional Reconstruction, China International Neuroscience Institute (CHINA-INI), Beijing, China; 5 National Center for Neurological Disorders, Beijing, China; 6 Department of Neuro-oncology, Cancer Center, Beijing Tiantan Hospital, Capital Medical University, Beijing, China; 7 Capital Medical University, Beijing, China

**Keywords:** Posttraumatic syringomyelia, Spinal cord injury, Cerebrospinal fluid, Intraspinal pressure, Bypass, Hydrocephalus

## Abstract

**Objective:**

Recent discoveries focused on the role of intraspinal pressure (ISP) in metabolite clearance after spinal cord injury (SCI) have initiated intense research on CSF inflow and outflow pathways. This study aimed to investigate whether the pulsatile ISP wave serves as the primary driver of posttraumatic syringomyelia (PTS) formation and progression, and to determine if a novel subarachnoid‑subarachnoid (S‑S) bypass procedure can effectively attenuate this abnormal pulsatile ISP wave in affected PTS patients.

**Methods:**

In this prospective cohort study of patients with PTS, ISP across the injury site was monitored intraoperatively both before and after placement of the S-S bypass tube. Neurological impairment was assessed using standardized scales at baseline, one year postoperatively, and at the final follow-up. A logistic regression model was used to analyze prognostic factors associated with surgical outcomes.

**Result:**

All 64 enrolled patients underwent S-S bypass surgery; 54 (84.4%) had a history of incomplete SCI. After a mean follow-up of 23.6 months (range: 12–36 months), 46 patients (71.9%) showed neurological improvement. Multivariate logistic regression identified three independent predictors of positive recovery: the pre-bypass ISP gradient across the injury site (OR = 2.228, P = 0.036), the pre-bypass ISP amplitude measured cranial to the injury site (OR = 4.269, P = 0.031) and a bypass tube spanning vertebral levels (OR = 0.331, P = 0.020). Receiver operating characteristic (ROC) curve analysis determined the optimal predictive cut-offs for these parameters. A pre-bypass ISP gradient > 1.5 mmHg showed a sensitivity of 93.48% and a specificity of 66.67%, a pre-bypass ISP amplitude measured cranial to the injury site >3.5 mmHg yielded a sensitivity of 82.61% and a specificity of 94.44%, while a bypass spanning < 5.5 vertebral levels showed a sensitivity of 100% and a specificity of 44.44%.

**Conclusions:**

S-S bypass procedure can effectively reduce abnormal Pulsatile Pressure Wave in patients with PTS. This study demonstrates that a pre-bypass ISP gradient > 1.5 mmHg across the injury site, a pre-bypass ISP amplitude >3.5 mmHg measured cranial to the injury site and a bypass conduit spanning < 5.5 vertebral levels are strong, independent predictors of neurological recovery after S-S bypass surgery for posttraumatic syringomyelia.

**Supplementary Information:**

The online version contains supplementary material available at https://doi.org/10.1186/s12987-026-00846-x.

## Introduction

Recent glymphatic system advances have renewed interest in cerebrospinal fluid (CSF) dynamics, particularly regarding spinal CSF inflow and outflow pathways [1–3]. Spinal cord injury (SCI) is a relatively common condition with an incidence of approximately 4.5 per 100,000 person-years and is associated with significant morbidity and mortality [4]. Posttraumatic syringomyelia (PTS) is a serious complication characterized by progressive neurological deterioration caused by a CSF-filled syrinx within the spinal cord following SCI and obstructed CSF circulation, resulting in devastating consequences for affected patients and often leading to poor neurological outcomes [5–7]. The exact pathophysiology of PTS remains incompletely understood [5]. Several theories have been proposed, including the water hammer theory, the craniospinal pressure dissociation theory, venous congestion of the spinal cord, the intramedullary pulse pressure theory, the bulk flow theory, and disruption of the blood–spinal cord barrier or other molecular barriers. No single theory fully explains all clinical and radiological observations, and the relative contribution of each mechanism likely varies among patients. Among these, the bulk flow theory – which postulates that a pressure gradient drives fluid from the subarachnoid space into the spinal cord – has gained considerable support, although it is not universally accepted [1,3, 8–11, 12].

Current treatment strategies for syringomyelia focus on restoring physiological CSF pathways by reconstructing spinal subarachnoid channel where feasible [9, 13, 14]. While craniovertebral decompression achieves favorable outcomes in hindbrain-related [12] and post-syringobulbia cases [3], the management of primary obstructive syringomyelia—often via laminectomy with duraplasty or shunting—is associated with comparatively lower success rates [3, 15]. To address this challenge, our team previously developed a novel minimally invasive subarachnoid-subarachnoid (S-S) bypass technique [9], which we have since successfully applied to other etiologies, including non-traumatic postoperative adhesive syringomyelia [16] and inflammation related syringomyelia.

**Fig. 1:**
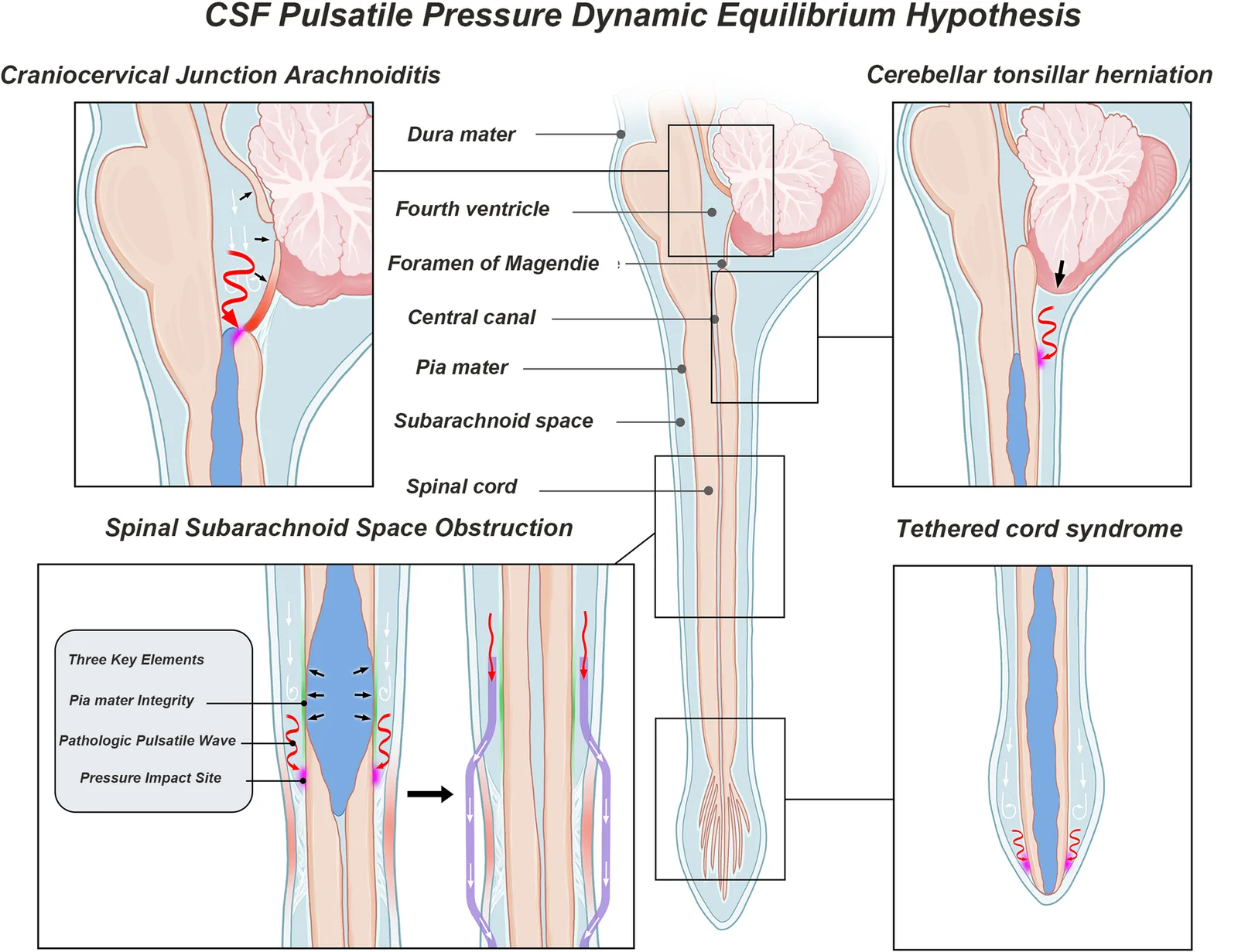
Schematic overview of the pathophysiological mechanisms driving syringomyelia across diverse etiologies. This schematic depicts both the normal anatomical structures and the pathological alterations implicated in syringomyelia formation. The normal anatomy comprises the fourth ventricle, foramen of Magendie, central canal, pia mater, dura mater, subarachnoid space, and spinal cord. The pathological conditions shown include cerebellar tonsillar herniation (e.g., Chiari malformation), craniocervical junction arachnoiditis, spinal subarachnoid space obstruction (pre‑ and post‑S‑S bypass), and tethered cord syndrome—all of which contribute to impaired CSF dynamics. Three key elements are identified as essential for syringomyelia pathogenesis: (1) pia mater integrity, (2) pathologic pulsatile wave, and (3) pressure impact site. Together, these elements create a self‑sustaining dynamic equilibrium, in which fluid is propelled into the spinal cord parenchyma, accumulates, and is prevented from egress by an intact pia mater, thereby perpetuating syringomyelia formation and progression

**Fig. 2:**
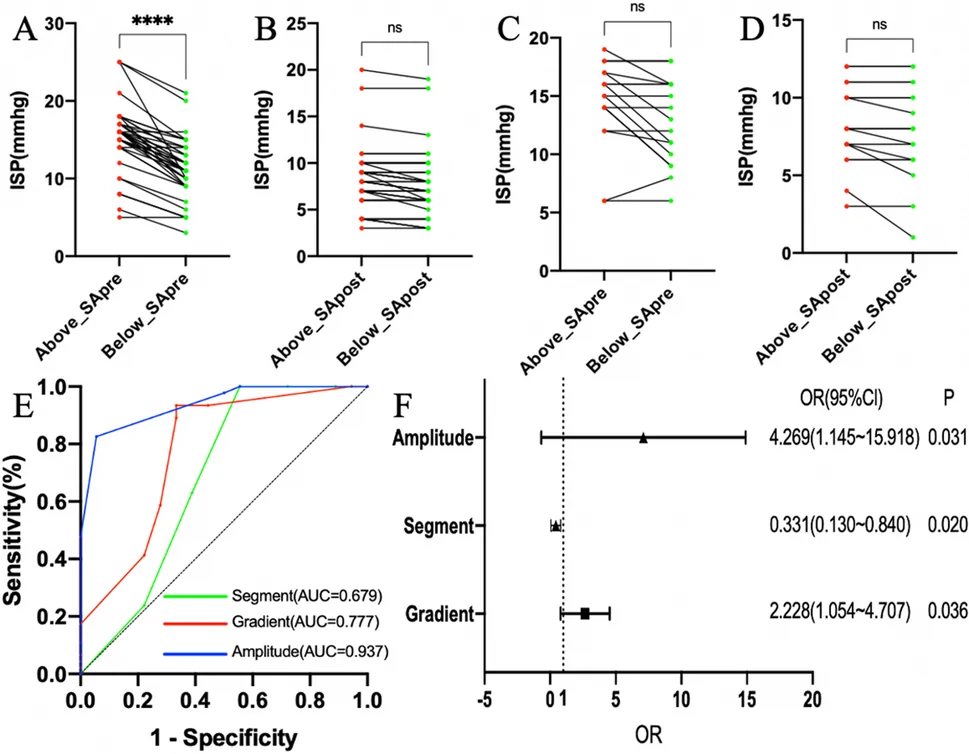
Comparative analysis of ISP gradients in favorable versus unfavorable outcome subgroups. (**A**, **B**) Favorable Outcome Subgroup: (**A**) Preoperative pressure analysis revealed a significant craniocaudal gradient across the sub-arachnoid adhesion. (**B**) Post-shunting tracings show normalization of this gradient. (**C**, **D**) Unfavorable Outcome Subgroup: (**C**) Preoperative analysis indicated no significant baseline pressure gradient across the sub-arachnoid adhesion. (**D**) Post-shunting tracings show without obvious change of this gradient. Predictive modeling for neurological recovery in post-traumatic syringomyelia. (**E**) Receiver operating characteristic (ROC) curves were used to evaluate the discriminant capacity of the ISP gradient, ISP amplitude measured cranial to the injury site and bypass span. (**F**) Forest plot from the multivariate logistic regression analysis identifying ISP gradient, ISP amplitude measured cranial to the injury site and bypass span as independent predictors of outcome. OR, Odds ratio. AUC, Area under the curve. SA: Sub-arachnoid adhesion

Nevertheless, the precise mechanism underlying the clinical efficacy of the S-S bypass remains unclear [9]. Unlike ventriculoperitoneal shunting for hydrocephalus, which creates an alternative, low-pressure drainage pathway [9], the S-S bypass aims to reconstruct physiological CSF flow within the subarachnoid space. This raises a fundamental question: how does the procedure facilitate clinical improvement. Previous studies on syringomyelia associated with Chiari malformation have indicated that the pulse‑pressure generated by the piston effect is a key factor in syrinx formation, and that its reduction may correlate with syrinx regression. We hypothesize that in PTS secondary to SCI, CSF flow is obstructed at the injury site, accompanied by elevated pulse pressure, thereby generating high‑amplitude pressure waves. Concurrently, the low compliance of the spinal cord at the adhesive site prevents energy dissipation through deformation, rendering it a "hard‑landing" zone where the pulse wave cannot be effectively attenuated. This high‑amplitude pressure wave acts like a fluid pump, continuously propelling fluid bulk flow into the fragile and loose spinal cord parenchyma at the adhesion site. Meanwhile, the intact pia mater impedes fluid egress, allowing the syrinx to maintain a dynamic equilibrium under high‑tension conditions. Furthermore, if the S-S bypass functions by re-establishing laminar CSF flow governed by Poiseuille’s law [17], the length of the bypass conduit—given the anatomically constrained, fixed diameter (internal diameter 1.1 mm, external diameter 2.5 mm, Sophysa Systems Corporation) within the spinal canal [9]—becomes a critical determinant of its hemodynamic efficacy and ability to restore sufficient flow (Fig. 1 and 2).

To address these questions, we conducted a prospective cohort study utilizing our minimally invasive S-S bypass technique. We systematically measured ISP both cranial and caudal to the injury site before and after bypass tube placement and performed regression analyses to correlate these parameters with clinical outcomes, with the aim of refining surgical strategy and improving patient prognosis.

## Method

### Study sample

This study included 64 consecutive patients (supFigure 1) with symptomatic PTS treated at the Xuanwu hospital and First Hospital of Hebei Medical University between Jan 2022 and 2025.

All patients had progressive neurological symptoms and underwent S–S bypass. The inclusion criteria were as follows: (1) history of traumatic SCI; (2) evidence of progressive neurological deficit, and/or pain syndrome; (3) CSF‑filled cavity within the spinal cord, clearly visible on T2‑weighted MRI, occurring at or adjacent to a prior SCI site, regardless of any history of prior syringomyelia intervention; and (4) minimum follow-up period of more than 1 year. Patients with only myelomalacia, intradural cyst, or syringomyelia due to other spinal diseases (such as spinal infection, tuberculous meningitis, or tumor) were excluded from this study.

All participants provided written informed consent before the surgery, and our institutional review board approved the study ([2025]073-003).

### Clinical and radiological assessments

The preoperative status and postoperative clinical course were assessed according to several grading systems: the Frankel grading system [18] for global neurological status and the American Spinal Injury Association (ASIA) motor and sensory score for motor weakness or sensory disturbance [19]. The results of surgical treatment were assessed by comparing the preoperative and postoperative values of each score. The major presenting symptoms or signs were assessed by the patients in terms of symptom improvement, stabilization, or deterioration (subjective grading) [20].

All patients underwent preoperative MRI and postoperative MRI, 3months, 1 year and annually 1 year. Preoperative and postoperative MR images were used to analyze the size and the craniocaudal extension of the syrinx. The sizes and widths of the syrinx were measured according to those of the T1 low-intensity areas. Improvement was defined as a reduction in preoperative neurological deficits and/or subjective report of symptoms by the patient whose deficits had improved and whose syrinx length, maximal cross-sectional diameter, or both decreased.

All measurements were performed by 2 investigators blinded to the name, clinical findings, and other imaging data of the patient.

### Surgical methods

The patient was positioned prone under general anesthesia. Using preoperative MRI and myelography to identify the normal subarachnoid spaces above and below the injury site, a limited fenestration was performed at one or two laminae to expose intact dura. If the planned incisions were proximate, a single midline incision was used to preserve the bone, scar tissue, and dura overlying the adhesion. After exposing normal dura at both cephalad and caudad sites, a subcutaneous tunnel was created using a tunneler. Two medical-grade silicone tubes (internal/external diameter: 1.1/2.5 mm, Sophysa Systems Corporation) were then passed through this tunnel. Under surgical microscope guidance, a ∼3 mm dural incision was made bilaterally on either side of the midline at the cranial site, and the arachnoid was opened. The proximal ends of the tubes were advanced approximately 5 cm into the normal subarachnoid space. The same procedure was repeated at the caudal site. Side holes are present at both the proximal and distal ends of the shunt tube. No arachnoid dissection was performed at the scarred injury site. Correct intrathecal placement was confirmed by observing CSF flow and bubble movement within the transparent tubes. Patency of the bypass was further verified by injecting sterile saline through one tube and observing return flow in the other. Finally, the dura was closed watertight with 6 − 0 non-absorbable sutures, and the tubes were secured to the dura and overlying fascia with 4 − 0 non-absorbable sutures to prevent displacement. The tunneled segment of both catheters remained in the subcutaneous space. The wound was closed in layers.

### Measurement of ISP

**Fig. 3:**
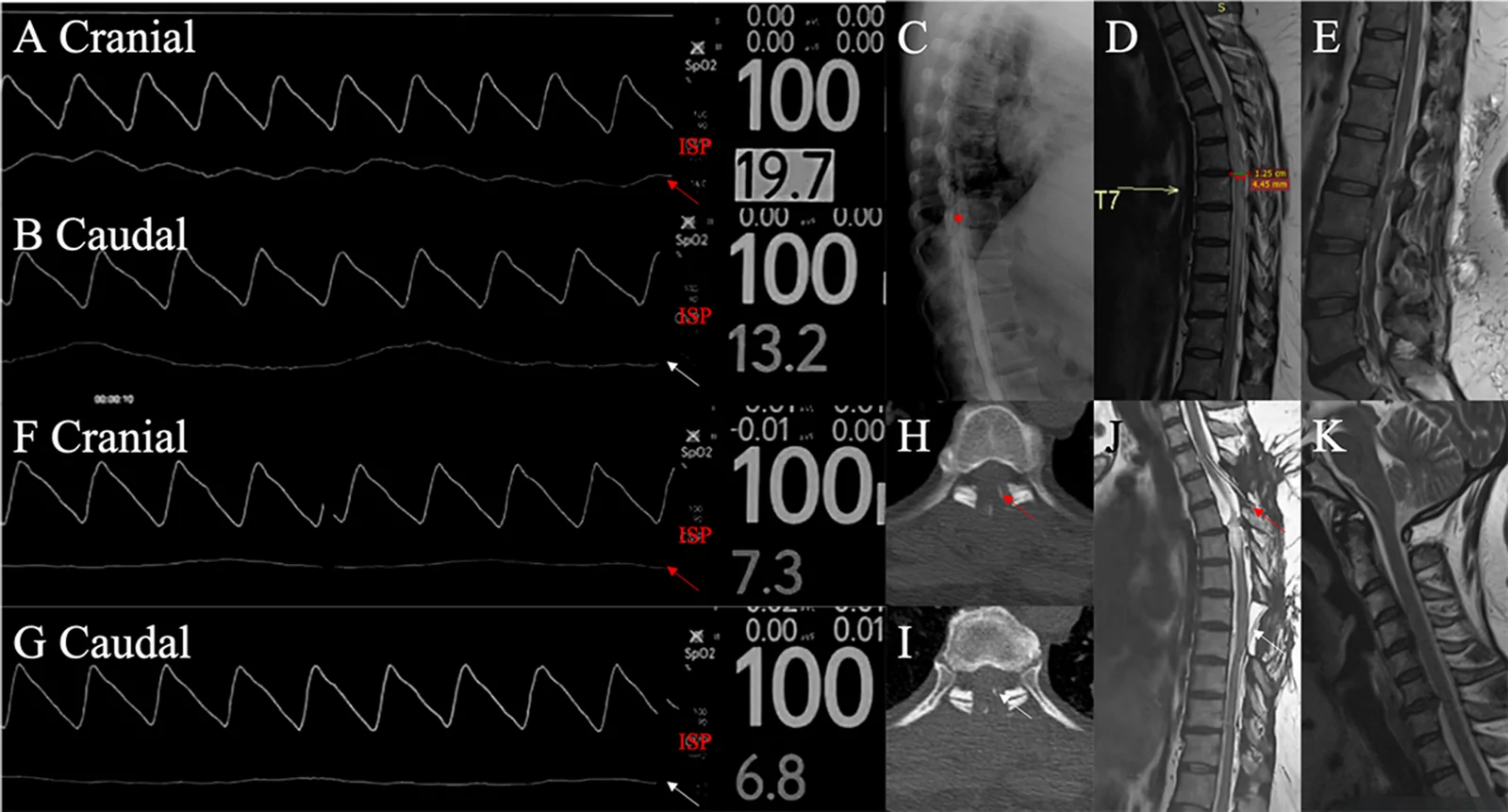
A 40-year-old female presented with progressive numbness and weakness in both lower extremities for over one year following a thoracic SCI, culminating in an inability to walk over the preceding three months. CASE1: (**A**, **B**) Preoperative intraspinal pressure (ISP) tracings cranial (**A**, red arrow) and caudal (**B**, white arrow) to the arachnoid scar show markedly elevated pressure gradient with superimposed abnormal waveform patterns. (**C**) Lumbar myelogram demonstrating a complete block at the T7 level due to dorsal arachnoid adhesion (red asterisk). (**D**, **E**) Preoperative T2-weighted magnetic resonance (MR) images reveal an intramedullary syrinx and spinal cord edema extending rostrally to T5. (**F**, **G**) Postoperative ISP tracings at corresponding locations (**F**: cranial, **G**: caudal) after S-S bypass placement demonstrate normalization of cranial waveforms and a significant reduction in pressure gradient. (**H**, **I**) Postoperative computed tomography (CT) images confirm the position of the S-S bypass conduit spanning from T4 (red arrow) to T7 (white arrow). (**J**, **K**) Postoperative T2-weighted MR images show marked regression of the syrinx and spinal cord edema. Supplementary video 1 shows the patient walking again after S-S bypass

**Fig. 4:**
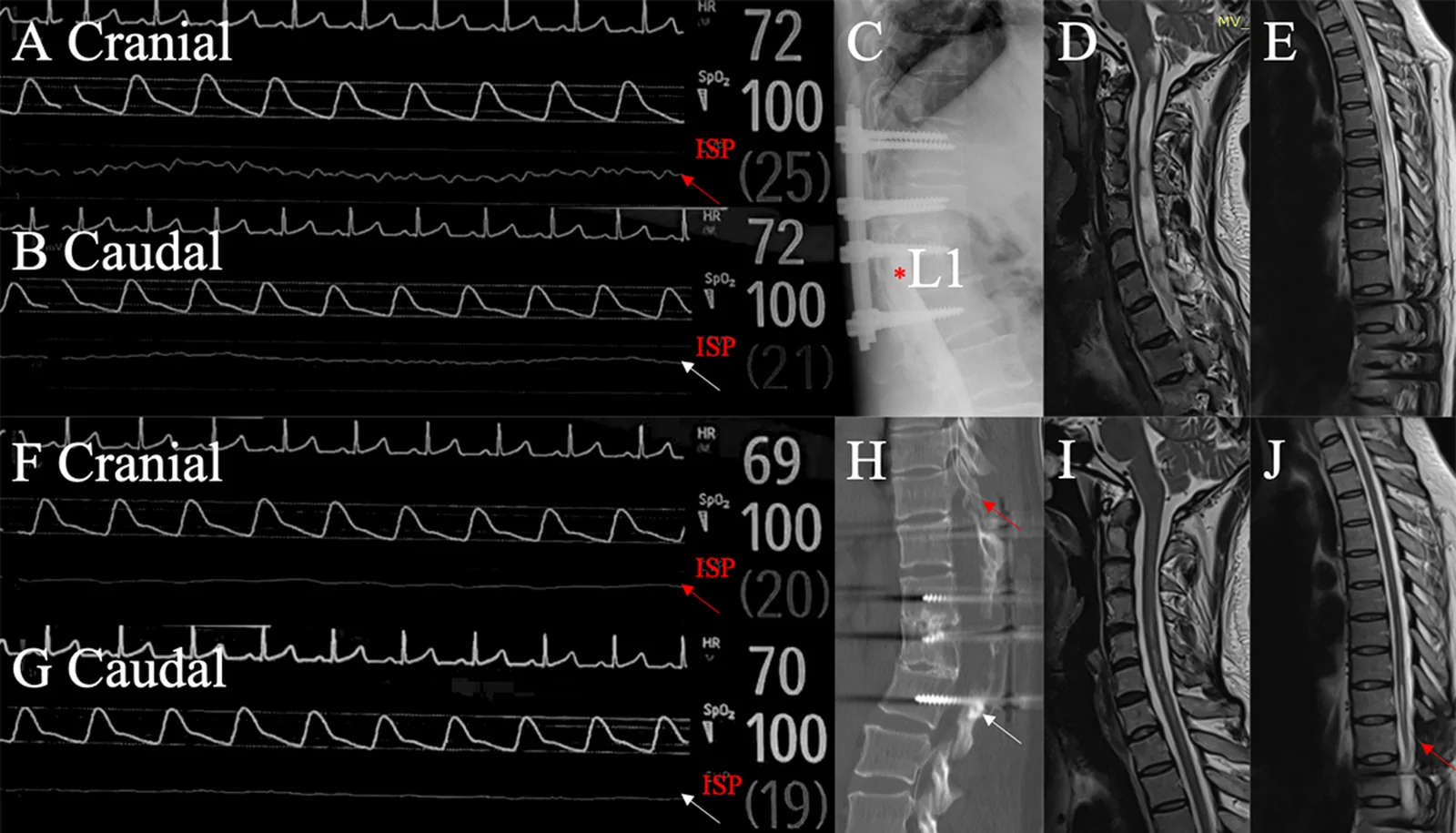
A 30-year-old male presented with pain and numbness in the left upper extremity for two years. His history included a SCI at the T12–L1 level sustained in a traffic accident fifteen years prior. CASE2: (**A**, **B**) Preoperative intraspinal pressure (ISP) tracings cranial (**A**, red arrow) and caudal (**B**, white arrow) to the arachnoid adhesion at L1 demonstrate elevated pressure gradient with abnormal waveform morphology. (**C**) Lumbar myelogram confirming a complete subarachnoid block at the L1 level (red asterisk) due to dorsal arachnoid scarring. (**D**, **E**) Preoperative T2-weighted magnetic resonance (MR) images reveal a large syrinx extending rostrally to the medulla oblongata. (**F**, **G**) Postoperative ISP tracings at corresponding locations (**F**: cranial, **G**: caudal) after S-S bypass placement show stable waveforms without a significant change in the pressure gradient. (**H**) Postoperative computed tomography (CT) localizer image confirming the position of the S-S bypass conduit spanning from T11 (red arrow) to L2 (white arrow). (**I**, **J**) Postoperative T2-weighted MR images demonstrate marked regression of the syrinx

**Fig. 5:**
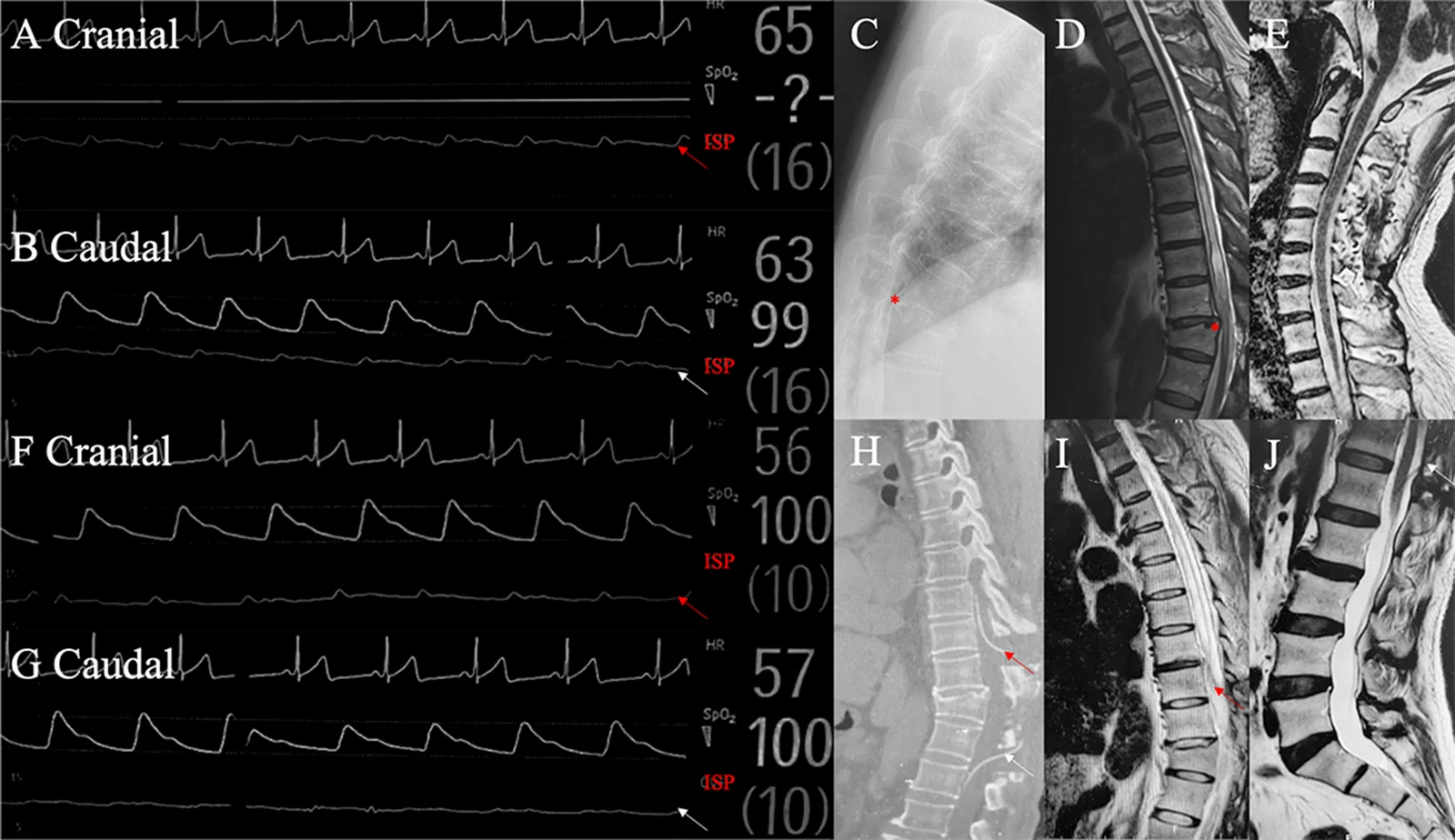
A 60-year-old male presented with progressive leg motor decline over two years, two decades after sustaining a T11-level SCI in a traffic accident. CASE3: (**A**, **B**) Preoperative intraspinal pressure (ISP) tracings cranial (**A**, red arrow) and caudal (**B**, white arrow) to the arachnoid adhesion at T11 show no abnormal pulsatile ISP wave. (**C**) Lumbar myelogram confirming a complete subarachnoid block at the T11 level (red asterisk) corresponding to dorsal arachnoid scarring.(**D**, **E**) Preoperative T2-weighted magnetic resonance (MR) images demonstrate an intramedullary syrinx extending rostrally to T5. (**F**, **G**) Postoperative ISP tracings at corresponding locations (**F**: cranial, **G**: caudal) following S-S bypass placement show stable waveforms without a significant change in the pressure gradient. (**H**) Postoperative computed tomography (CT) localizer image confirming the position of the S-S bypass conduit spanning from T10 (red arrow) to L1 (white arrow). (**I**, **J**) Postoperative T2-weighted MR images showed no change in the syrinx morphology

Intraspinal pressure (ISP) was measured with the patient in the prone position under general anesthesia with mechanical ventilation. Prior to shunt placement, a catheter (outer diameter, 0.9 mm) was inserted to measure ISP at both the cranial and caudal ends of the arachnoid adhesion. ISP was transduced using a non-flush strain-gauge transducer attached to non-pressurized tubing filled with saline solution (Figs. 3, 4 and 5). The dorsal surface of the spinal cord served as the reference plane for all measurements. Mean ISP was recorded during the expiratory pause between respirations. For each location, continuous pressure waveforms were recorded for ≥ 3 min during stable respiration and heart rate. Recordings with visible movement or breathing artefacts were discarded.

Following baseline recordings, the catheter was opened to allow spontaneous CSF drainage. Post-drainage pressures at both the cranial and caudal ends were recorded immediately after one bypass tube implantation. All measurements were performed independently by two neurosurgeons. The ISP gradient was defined as the difference between the ISP measured proximal (above) the site of subarachnoid obstruction and the ISP measured distal (below) the obstruction. The cranial ISP pulse amplitude was defined as the mean peak-to-trough pressure difference (P_peak - P_trough) across all cardiac cycles recorded cranial to the obstruction during at least three consecutive artefact-free respiratory cycles.

### Statistical analysis

Analyses were conducted using SPSS software (version 26, IBM Corp) and Prism (version 9.0, LLC). We used the Fisher exact test, the Pearson χ^2^ test, for categorical variables, the nonparametric Mann-Whitney U test, and the Student t test or one-way analysis of variance test for continuous variables. Multivariable analysis was performed using logistic regression, including factors with a p‑value < 0.05: bypass tube length (as a continuous variable), pressure gradient (as a continuous variable), ISP amplitude measured cranial to the injury site (as a continuous variable) and lumbosacral outlet obstruction (presence/absence).Odds ratios and 95% CIs were calculated for favorable outcomes. Receiver operating characteristic curves were calculated to investigate the diagnostic value of biomarkers. All P-values were two-sided, and we defined statistical significance as *P* < 0.05.

### Result

Our cohort comprised 64 patients (46 male, 18 female) who underwent S-S bypass for posttraumatic syringomyelia, with a mean age of 46.8 years (range: 6–74). The median time from original SCI to PTS surgery was 166.0 months(range:12–480); and the median time from MRI diagnosis of syringomyelia to surgery was 35.8 months (range:1–240). As detailed in Table 1, the majority of cases involved incomplete SCI (84.4%) at the thoracic or lumbar level. Furthermore, most patients received S-S bypass conduit spanning no more than 5 vertebral levels (87.5%). Postoperatively, clinical improvement was observed in 46 patients (71.9%), while 18 patients (28.1%) maintained a stable neurological status. Radiological evaluation demonstrated syrinx reduction in 46 patients, with the remaining 18 exhibiting stable morphology. Complications occurred in 3.2% of cases (*n* = 2), manifesting as aseptic meningitis in both instances, which resolved spontaneously within two weeks. No cases of CSF leakage requiring intervention were recorded. The mean follow-up duration was 23.6 months (range: 12–36).

**Table 1:** Clinical findings, MRI, and surgical outcome findings in posttraumatic syringomyelia group

	Posttraumatic syringomyelia (*n* = 64)
**Male**	46(71.9%)
**Age**	46.8 ± 12.6
**Previous ASIA**	
Complete	10(15.6%)
Incomplete	54(84.4%)
**Previous surgery**	
With	50(78.1%)
Conservative	14(21.9%)
**Interval (mos) between first SCI and PTS surgery**	166.0 ± 122.3
**PTS Symptom duration (mos)**	35.8 ± 40.4
**Symptoms**	
Neuropathic pain	34(53.1%)
Dysesthesia	64(100%)
Hypesthesia	64(100%)
Motor power	55(85.9%)
Gait	45(70.30%)
Sphincter function	52(81.3%)
**ASIA sensory**	183.3 ± 30.1
**ASIA motor**	79.5 ± 19.3
**Radiographic**	
**Previous injury location**	
Cervical	2(3.1%)
Thoracic	44(68.8%)
Lumbar	18(28.1%)
**Syrinx length(levels)**	13.8 ± 5.9
**Syrinx location**	
Medulla oblongata	15(23. 4%)
Cervical	1(1.6%)
Cervicothoracic	46(71.90%)
Thoracic	17(26.5%)
Syrinx/canal*	
A	49(76.6%)
B	14(21.8%)
C	1(1.6%)
**Pressure gradient**	
With	50(78.1%)
Without	14(21.9%)
**ISP pulse amplitude (peak-to-trough) measured cranial to the injury site(mmHg)**	3.7 ± 1.4
**Bypass segment**	
≤ 5	56(87.5%)
> 5	8(12.5%)
**Lumbosacral outlet obstruction**	
Without	60(93.8%)
With	4(6.2%)
**Outcome**	
Favorable	46(71.9%)
Unfavorable	18(28.1%)
**Complication**	
Neurological disorders	0
Aseptic meningitis	2(3.2%)
CSF fistula	0(0)
Isolated fever	0(0)
Pneumonia	0(0)
**Follow-up(mos)**	23.6 ± 6.2

*Syrinx/Spinal canal: maximal diameter of syrinx/spinal canal. A:75%-100%, B 50–75%, C < 50%

There were no significant baseline clinical differences between the favorable and unfavorable outcome groups (Table 2) in terms of ASIA impairment scale grade (*P* = 0.599) or syrinx location (*P* = 0.102). However, the favorable outcome group exhibited significant differences in three key intraoperative parameters: a higher-pressure gradient across the injury site (*P* < 0.05), a higher-pressure pulse amplitude (peak‑to‑trough) measured cranial to the injury site (P < 0.05) and a shorter bypass conduit span (*P* < 0.05). Additionally, the presence of lumbosacral outlet obstruction was significantly different between the two groups (*P* = 0.005).

**Table 2:** Clinical, MRI, and outcome data of post-traumatic syringomyelia patients with favorable and unfavorable outcome after minimally invasive S-S bypass

	Favorable PTS (*n* = 46)	Unfavorable PTS (*n* = 18)	*P*
**Male**	34(73.9%)	12(66.7%)	0.562
**Age**	47.3 ± 10.2	45.6 ± 17.1	0.632
**Previous ASIA**			0.599
Complete	6(13.0%)	4(22.2%)	
Incomplete	40(87.0%)	14(77.8%)	
**Previous surgery**			1.000
With	36(78.3%)	14(77.8%)	
Conservative	10(21.7%)	4(22.2%)	
**Interval (mos) between first SCI and PTS surgery**	159.3 ± 121.6	183.1 ± 122.7	0.492
**PTS Symptom duration (mos)**	40.2 ± 45.1	24.3 ± 20.6	0.162
**Symptoms**			
Neuropathic pain	26(56.5%)	8(44.4%)	0.384
Dysesthesia	46(100%)	18(100%)	1.000
Hypesthesia	46(100%)	18(100%)	1.000
Motor power	40(87.0%)	15(83.3%)	1.000
Gait	33(71.7%)	12(66.7%)	0.690
Sphincter function	36(78.3%)	16(88.9%)	0.533
**ASIA sensory**	186.8 ± 25.9	174.4 ± 37.4	0.143
**ASIA motor**	81.4 ± 18.8	74.6 ± 20.0	0.215
**Radiographic**			
**Previous injury location**			0.482
Cervical	1(2.2%)	1(5.6%)	
Thoracic	33(71.7%)	11(61.1%)	
Lumbar	12(26.1%)	6(33.3%)	
**Syrinx length(levels)**	14.3 ± 5.5	12.5 ± 6.5	0.272
**Syrinx location**			0.102
Cervical	1(2.2%)	0(0)	
Cervicothoracic	36(78.3%)	10(55.6%)	
Thoracic	9(19.5%)	8(44.4%)	
Syrinx/canal*			0.653
A	36(78.3%)	13(72.2%)	
B	9(19.6%)	5(27.8%)	
C	1(2.1%)	0	
**Pressure gradient**	4.217 ± 2.375	1.500 ± 2.307	**< 0.001**
With	44(95.7%)	6(33.3%)	
Without	2(4.3%)	12(66.7%)	
**ISP pulse amplitude (peak-to-trough) measured cranial to the injury site(mmHg)**	4.4 ± 1.0	2.1 ± 1.1	**< 0.001**
**Bypass segment**	4.130 ± 0.778	5.167 ± 1.948	**0.003**
≤ 5	46(100%)	10(55.6%)	
> 5	0(0)	8(44.4%)	
**Lumbosacral outlet obstruction**			**0.005**
With	0(0)	4(22.2%)	
Without	46(100%)	14(77.8%)	
**Complication**			1.000
Neurological disorders	0	0(0)	
Aseptic meningitis	2(4.3%)	0(0)	
CSF fistula	0(0)	0(0)	
**Follow-up(mos)**	24.2 ± 5.9	22.0 ± 6.5	0.203

*Syrinx/Spinal canal: maximal diameter of syrinx/spinal canal. A:75%-100%, B 50–75%, C < 50%

Preoperative pressure analysis (Fig. 2) in the favorable outcome subgroup revealed a significant craniocaudal gradient across the arachnoid adhesion (cranial: 15.4 ± 4.2 mmHg vs. caudal: 11.1 ± 3.6 mmHg; *P* < 0.05), which normalized postoperatively (cranial: 8.2 ± 3.3 mmHg vs. caudal: 7.8 ± 3.3 mmHg; *P* > 0.05). In the unfavorable outcome subgroup, the preoperative gradient was less pronounced (cranial: 14.6 ± 3.7 mmHg vs. caudal: 13.1 ± 3.8 mmHg; *P* > 0.05), and postoperative reduction of cranial and caudal pressure was also observed (cranial: 8.2 ± 2.6 mmHg vs. caudal: 7.8 ± 3.0 mmHg; *P* > 0.05).

Multivariate logistic regression (Fig. 2) identified three independent predictors of positive neurological recovery: an ISP gradient across the injury site (OR = 2.228, P = 0.036), ISP amplitude measured cranial to the injury site (OR = 4.269, P = 0.031) and a bypass tube spanning vertebral levels (OR = 0.331, P = 0.020). Receiver operating characteristic (ROC) curve analysis established the optimal predictive cut-offs for these parameters. An ISP gradient > 1.5 mmHg demonstrated a sensitivity of 93.48% and a specificity of 66.67%, ISP amplitude measured cranial to the injury site >3.5 mmHg yielded a sensitivity of 82.61% and a specificity of 94.44%, while a bypass span of < 5.5 vertebral levels showed a sensitivity of 100% and a specificity of 44.44%.

## Illustrative cases

### Successful S-S bypass with abnormal pulsatile ISP wave

#### Case 1

A 40-year-old female presented with progressive numbness and weakness in both lower extremities for over one year following a thoracic SCI, culminating in an inability to walk over the preceding three months. Preoperative magnetic resonance imaging (MRI) demonstrated spinal cord edema and syrinx from T5 to T10. Intraoperative measurement prior to bypass placement revealed a significant ISP gradient of 6.5 mmHg. Two bypass conduits were placed from T4 to T7. Postoperatively, the patient’s clinical symptoms improved, including the recovery of walking ability. Independent ambulation was maintained at the final two-year follow-up. Follow-up MRI confirmed the resolution of the spinal cord edema and syrinx (Fig. 3).

### Case2

A 30-year-old male presented with pain and numbness in the left upper extremity for two years. His history included a SCI at the T12–L1 level sustained in a traffic accident fifteen years prior. MRI revealed a large intramedullary syrinx extending rostrally to medulla oblongata. A preoperative ISP gradient of 4 mmHg was recorded. Two bypass conduits were inserted, spanning from T11 to L2. Following the procedure, his symptoms showed improvement. Postoperative MRI demonstrated significant regression in both the length and width of the syrinx (Fig. 4).

### Failed S-S bypass without a significant abnormal pulsatile ISP wave

#### Case 3

A 60-year-old male presented with progressive leg motor decline over two years, two decades after sustaining a T11-level SCI in a traffic accident. MRI revealed an intramedullary syrinx extending to T5. Notably, no significant abnormal pulsatile ISP wave was detected prior to intervention. Despite the placement of two bypass conduits from T10 to L1, his symptoms only stabilized without improvement. Postoperative MRI showed no change in the syrinx morphology (Fig. 5).

### Failed S-S bypass with an excessively long conduit span

#### Case 4

**Fig. 6:**
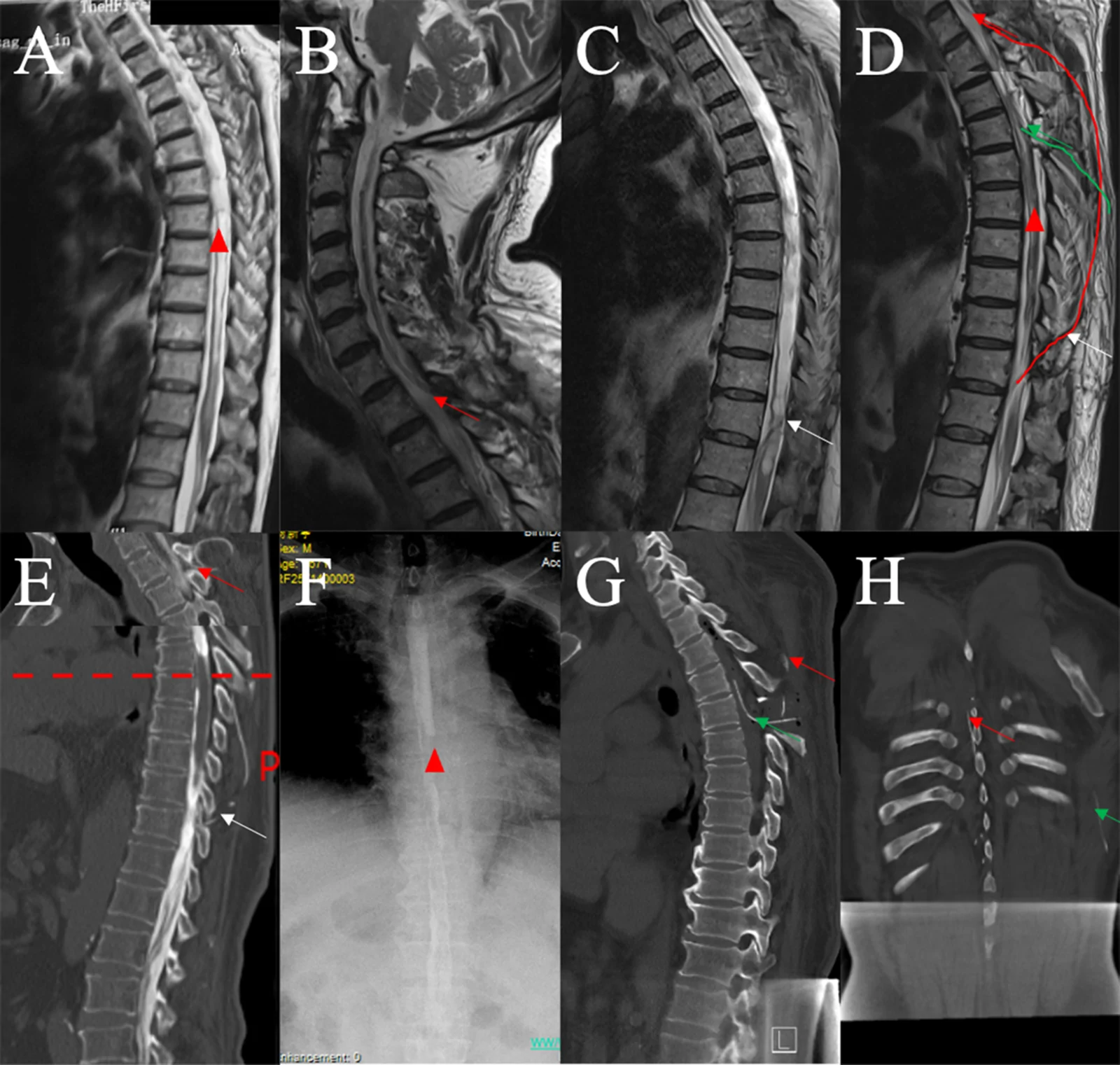
A 60-year-old male, who had undergone an S-S bypass from T2 to T11 two years earlier, presented with progressive lower extremity numbness, weakness, and a one-year history of gait instability. CASE4: (**A**) Preoperative T2-weighted magnetic resonance (MR) image demonstrates a large syrinx(red triangle) extending from T2 to T12. (**B**, **C**) Follow-up T2-weighted MR images obtained 2 years after S-S bypass (from T2 to T11, red and white arrows) show a persistent, stable syrinx. (**D**) A subsequent T2-weighted MR image following the addition of a thecal-peritoneal shunt (green arrow) reveals a significant reduction in syrinx size. (**E**, **F**) Postoperative CT myelography (**E**) and correlative X-ray (**F**) after lumbar contrast injection demonstrate patent, unobstructed contrast flow within the spinal canal following the initial S-S bypass procedure (T2 to T11, red and white arrows). (**G**, **H**) Postoperative computed tomography (CT) images confirm the final position of both the S-S bypass conduit (from T2 to T11, red and white arrows) and the T4-peritoneal shunt catheter (green arrow)

A 60-year-old male, who had undergone an S-S bypass from T2 to T11 two years earlier, presented with progressive lower extremity numbness, weakness, and a one-year history of gait instability. MRI showed a persistent syrinx. Due to the failed S-S bypass, a thecal-peritoneal shunt (set at 110 mmH₂O) was placed from T4. Postoperative follow-up confirmed regained ambulatory function and a significant reduction in syrinx size(Fig. 6).

## Discussion

These findings in PTS parallel the well-established relationships between ICP, cerebral perfusion pressure (CPP), and neurological recovery after SCI [1] or traumatic brain injury [21–24]. Spinal cord microdialysis studies suggest that elevated ISP and reduced spinal cord perfusion pressure (SCPP) contribute to injury site ischemia and secondary damage [8]. However, direct clinical evidence documenting the persistence of such pressure gradients following SCI has been lacking.

The precise biomechanical coupling mechanism driving the formation and progression of PTS have long been a subject of debate. Conventional theories have difficulty explaining why a progressive syrinx with a high‑tension state can develop and be sustained in patients with SCI, even when their mean ISP remains entirely normal. The experimental work [8] of Klekamp et al., characterizing ISP gradients in feline C2 injury models with dorsal scarring, provides a foundational concept. However, clinical translation is complicated by significant pathophysiological differences. Single-level obstructions in rat models [25] (except at specific sites like T13) often fail to generate sufficient hydrodynamic forces to drive substantial syrinx-forming fluid inflow, particularly when central canal communication persists. In contrast, human PTS typically involves complex, multilevel adhesions exacerbated by broader secondary injury cascades. These molecular responses [15] may facilitate CSF leakage into the central canal, potentially dissipating measurable pressure gradients and adding to the pathophysiological complexity. This may explain why the therapeutic potential of a straightforward, minimally invasive S-S bypass [9] to relieve pathological pressure gradients in PTS or other inflammatory syringomyelia remains underrecognized. Crucially, our S-S bypass technique provided a unique opportunity to directly measure the ISP gradient across the human injury site. Previous attempts to measure pressure across human scar tissue have been severely limited by surgical constraints. Our S-S bypass approach has enabled the first direct documentation of these gradients in a clinical setting, providing key evidence supporting our hypothesis, demonstrating that it is the pulsatile pressure wave—rather than static pressure—that serves as the original driving force and continuous energy source for syrinx formation within the spinal cord.

Early surgical decompression with duraplasty, typically within 24 h post-SCI, is known to reduce ISP and benefit neurological recovery [1]. Analogously, the historical gold standard for restoring CSF pathways in syringomyelia has involved decompression and duraplasty to surgically recreate the subarachnoid space [13]. While effective for limited focal arachnoidopathy, this open approach is high-risk for cases with extensive adhesions, carrying a significant likelihood of iatrogenic cord injury and adhesion recurrence [14]. Most patients with primary obstructive syringomyelia have a history of prior surgery and present with such extensive adhesions, rendering complete and safe arachnoid lysis exceedingly difficult. Alternative interventions, including percutaneous aspiration, myelotomy, or various non-physiological shunt procedures (e.g., syrinx-peritoneal or syrinx-pleural), fail to address the underlying obstructive pathophysiology [26]. These approaches are characterized by high revision rates and often poor success rates, with reported failure or complication rates of 50–97%, alongside poorly defined selection criteria.

In contrast, our minimally invasive S-S bypass technique directly addresses the core pathophysiology by reducing the pathological pressure wave to restore physiological CSF circulation within the spinal canal. Our analysis identifies the pressure differential across the injured segment as a critical risk factor. Placement of the bypass conduit alleviates this abnormal pulsatile ISP wave. Given the fixed spatial constraints of the spinal canal, the hemodynamic efficacy of a conduit with a fixed diameter (internal diameter 1.1 mm, external diameter 2.5 mm, Sophysa Systems Corporation) [9] is governed by Poiseuille’s Law, where fluid resistance is directly proportional to conduit length and inversely proportional to the fourth power of its internal radius. Therefore, to achieve sufficient flow to reduce ISP and restore circulation, the conduit must operate within an optimal length range. Our findings corroborate this principle, demonstrating that for a conduit of the specified fixed diameter, a length spanning less than 5.5 vertebral levels is necessary and sufficient to achieve the drainage volume required for clinical efficacy.

Resolution of the ISP gradient restores normal CSF dynamics across the obstructed subarachnoid space [8]. According to the Pulse Pressure Fluid Pump Dynamic Equilibrium Hypothesis, abnormal pulsatile ISP wave drive fluid from the subarachnoid space into the spinal cord parenchyma, thereby contributing to syrinx formation and expansion. Once this abnormal pulsatile ISP wave is eliminated by an S‑S bypass or a thecal‑peritoneal shunt, the driving force for transparenchymal fluid ingress is removed. Furthermore, restoration of laminar CSF flow enhances perivascular and interstitial clearance (i.e., the spinal cord ‘glymphatic’ system), allowing gradual reabsorption or drainage of the fluid accumulated within the central canal [27]. Over time, this leads to osmotic pressure homeostasis, which manifests as marked shrinkage of the syrinx on postoperative MRI.

Classical theories—Williams' craniospinal pressure dissociation and the systolic piston effect proposed by Oldfield and Heiss [5]—have laid the groundwork for understanding biomechanical coupling mechanism of syringomyelia. However, these models are largely constructed upon the specific anatomical substrate of Chiari malformation, with limited evidence available for other etiologies. The present study provides direct in vivo evidence of abnormal ISP waveforms in patients with PTS. Importantly, these aberrant waveforms are identified not only in spinal obstructive syringomyelia, but also in the fourth ventricle of classic communicating syringomyelia and the distal thecal sac of tethered cord-related syringomyelia. Taken together, these findings reveal a universal biomechanical mechanism applicable to all subtypes of syringomyelia. Three essential factors perpetuate high-tension syringomyelia: abnormal pulsatile pressure delivers continuous driving force, low-compliance, gray matter-enriched, loose focal anchoring sites act as the gateway for fluid infiltration into the spinal cord, and intact pia mater limits outward fluid drainage to maintain intramedullary hypertension, finally establishing a self-sustaining dynamic equilibrium [28–33]. This unified hypothesis carries direct clinical implications: future therapeutic strategies should be directed toward dissipating abnormal pulsatile energy, rather than being limited to adhesiolysis with duraplasty or syrinx shunting.

### Limitation

The follow-up period for S-S bypass in this study was relatively short (mean follow-up = 23.6 months (range: 12–36)). However, the primary objective was to introduce and establish a surgical indication framework and possible mechanism for this minimally invasive technique—similar to our previously reported mis-SSB procedure [9]. Furthermore, evidence is still needed to support the conclusions regarding “abnormal ISP waveforms,” which were based on very limited ISP recordings, without adequately excluding confounding factors such as anesthesia, respiration, or hemodynamics in our study. In addition, patients requiring multilevel bypass bridging generally have extensive long-segment arachnoid adhesions, and this feature may introduce bias to prognostic evaluation. Finally, with only 46 events, overfitting and lack of external validation is a major concern future large-scale, multicenter clinical trials will be essential to further validate our findings and to compare the S-S bypass technique to other treatments for post-traumatic syringomyelia [3, 9, 20, 34, 35].

## Conclusions

The S‑S bypass is a safe and effective procedure for post‑traumatic syringomyelia that avoids myelotomy and direct manipulation of adhesions. It eliminates the abnormal pulsatile ISP wave across the obstructed subarachnoid space, reduces syrinx size, and normalizes abnormal CSF accumulation. A preoperative ISP gradient > 1.5 mmHg, a pre-bypass ISP amplitude >3.5 mmHg measured cranial to the injury site and a bypass conduit spanning < 5.5 vertebral levels are strong independent predictors of neurological recovery.

## Supplementary Information

Below is the link to the electronic supplementary material.


Supplementary Material 1: SUP figure1: Flow chart for screening posttraumatic syringomyelia patients. 



Supplementary Material 2: SUP video1: Resume walking Following S-S Bypass in a Representative Patient with Posttraumatic Syringomyelia. Serial perioperative video documentation demonstrates the timeline of functional recovery. Preoperatively, the patient presented with paraplegia and significant lower limb edema. At one-week post-surgery, edema had substantially subsided, and nascent voluntary leg movement was observed. By one month, the patient achieved assisted ambulation using a walker, progressing to independent walking at the three-month assessment. Independent ambulation was maintained at the final two-year follow-up.


## Data Availability

The datasets supporting the conclusions of this article are available from the corresponding author on reasonable request.
